# Amelanotic Melanoma—Biochemical and Molecular Induction Pathways

**DOI:** 10.3390/ijms252111502

**Published:** 2024-10-26

**Authors:** Piotr Misiąg, Klaudia Molik, Monika Kisielewska, Paulina Typek, Izabela Skowron, Anna Karwowska, Jacek Kuźnicki, Aleksandra Wojno, Marcin Ekiert, Anna Choromańska

**Affiliations:** 1 Faculty of Medicine, Wroclaw Medical University, Pasteura 1, 50-367 Wroclaw, Poland; piotr.misiag@student.umw.edu.pl (P.M.); klaudia.molik@student.umw.edu.pl (K.M.); monika.kisielewska@student.umw.edu.pl (M.K.); paulina.typek@student.umw.edu.pl (P.T.); izabela.skowron@student.umw.edu.pl (I.S.); anna.karwowska@student.umw.edu.pl (A.K.); jacek.kuznicki@student.umw.edu.pl (J.K.); aleksandra.wojno@student.umw.edu.pl (A.W.); 2 Students Scientific Group No. 148, Faculty of Pharmacy, Wroclaw Medical University, 50-367 Wroclaw, Poland; 3 Department of Oncology, Wroclaw Medical University, pl. L. Hirszfelda 12, 53-413 Wroclaw, Poland; marcin.ekiert@umw.edu.pl; 4 Department of Molecular and Cellular Biology, Wroclaw Medical University, Borowska 211A, 50-556 Wroclaw, Poland

**Keywords:** amelanotic melanoma, melanoma, precision medicine, targeted therapy, immunotherapy, melanogenesis, biomarkers, prognostic factors

## Abstract

Amelanotic melanoma (AM) is a subtype of hypomelanotic or completely amelanotic melanoma. AM is a rare subtype of melanoma that exhibits a higher recurrence rate and aggressiveness as well as worse surveillance than typical melanoma. AM shows a dysregulation of melanin production, cell cycle control, and apoptosis pathways. Knowing these pathways has an application in medicine due to targeted therapies based on the inhibiting elements of the abovementioned pathways. Therefore, we summarized and discussed AM biochemical and molecular induction pathways and personalized medicine approaches, clinical management, and future directions due to the fact that AM is relatively rare. AM is commonly misdiagnosed. Hence, the role of biomarkers is becoming significant. Nonetheless, there is a shortage of biomarkers specific to AM. BRAF, NRAS, and c-KIT genes are the main targets of therapy. However, the role of BRAF and KIT in AM varied among studies. BRAF inhibitors combined with MAK inhibitors demonstrate better results. Immune checkpoint inhibitors targeting CTLA-4 combined with a programmed death receptor 1 (PD-1) show better outcomes than separately. Fecal microbiota transplantation may overcome resistance to immune checkpoint therapy of AM. Immune-modulatory vaccines against indoleamine 2,3-dioxygenase (IDO) and PD ligand (PD-L1) combined with nivolumab may be efficient in melanoma treatment.

## 1. Introduction

Amelanotic malignant melanoma (AMM) represents a subtype of melanoma characterized by hypomelanotic or completely amelanotic features [1]. Approximately 8% of all melanoma cases are identified as AMM, though this percentage varies across studies. Additionally, AMM appears to have a higher prevalence in pediatric patients [2]. AMM tumor cells are of melanocytic lineage and have the biological capacity to produce melanin. AMMs express microphthalmia-associated transcription factor (MITF) and tyrosinase. After a biopsy, pigment can be detected in less than 5% of tumor cells, but the pigmentation is invisible clinically and dermosopically, therefore raising problems with stating the correct diagnosis [3,4]. Most AMMs exhibit an epithelioid morphology, with additional variants presenting as spindled or desmoplastic forms [3]. Among other features, AMM is often described to exhibit a thicker Breslow thickness and a higher mitotic rate than pigmented melanoma [1]. Delayed diagnosis or misdiagnosis often leads to inappropriate/delayed treatment. The rapid tumor growth rate in the later stages of AMM, combined with described AMM features, results in lower survival rates of patients with AMM [5]. Both dermoscopy and reflectance confocal microscopy are accurate, high specificity, and moderate sensitivity techniques for diagnosing AMM [6]. Unfortunately, due to the lack or low levels of pigmentation and inconsistency with the “ABCD” criteria, the diagnosis of AMM is still often delayed [1]. Many cases of AMM are diagnosed in pediatric patients and for them, additional “ABCD” criteria are being proposed, including “A”, standing for “amelanotic” [7]. The ABCD scale is a widely used method for evaluating moles and assessing the risk of melanoma. Each letter in the acronym represents a different characteristic to look for: A (Asymmetry), B (Border), C (Color), and D (Diameter). This scale helps detect early melanoma by providing clear criteria for patients and healthcare providers to evaluate skin lesions. Regular skin examinations using the ABCD criteria can improve outcomes by identifying potentially malignant moles at an earlier stage. For amelanotic melanoma, which lacks pigment (appears flesh toned, pink, or red), the ABCD rule may be less valuable because these tumors do not exhibit the typical changes in color or pigment. Diagnosing amelanotic melanoma often requires more attention to subtle changes in size, shape, or elevation of the lesion and symptoms like itching or bleeding. Additional diagnostic tools, such as E for Evolution (any change over time), can also help identify these types of melanoma. While the ABCD rule is a helpful guideline for melanotic melanoma, its applicability is limited for detecting amelanotic melanoma due to the lack of pigment. More emphasis is needed on changes in the lesion’s shape, size, and evolving features [1,2,3]. Often in clinical practice, AMM is misdiagnosed with other benign or malignant lesions. To determine AMM in demanding cases, electron microscopy can be performed in order to detect melanosomes [4]. Histology and immunochemistry remain the golden standard for AMM diagnosis. Among immunochemical marker S100 is the most sensitive, others include Melan-A, HMB-45, tyrosinase, MITF, and Ki-67 [1]. Increasing our knowledge of molecular mechanisms associated with AMM could help differentiate AMM and pigmented melanoma and discover new AMM biomarkers. It has been reported that there are significant differences at the proteome level between AMM and pigmented melanoma [8]. It is crucial to elucidate the biochemical and molecular induction pathways of AMM and the dysregulation of melanogenesis. Understanding these mechanisms can significantly enhance recent advancements in the diagnosis and treatment of this melanoma subtype.

## 2. Molecular Characteristics of Amelanotic Melanoma

### 2.1. Absence of Melanin Pigment and Its Implications

The lack of melanin pigment in amelanotic melanoma is a crucial characteristic that significantly complicates the clinical diagnosis. AM can resemble other skin conditions, such as basal cell carcinoma, Bowen’s disease, eczema, keratoacanthoma, pyogenic granuloma, or extramammary Paget’s disease [9]. The absence of pigmentation can result in misdiagnosis or delayed diagnosis and treatment, hindering the application of color criteria in the ABCDE algorithm. Consequently, the prognosis of patients with AM is generally poorer than that of patients with pigmented melanoma, predominantly due to the more advanced stage at which it is diagnosed. Furthermore, AM exhibits more aggressive pathological features than pigmented melanoma, narrowing the window for early detection [2,10,11]. Additionally, patients with amelanotic metastases tend to have a worse prognosis than those with pigmented metastases [12].

### 2.2. Genetic Alterations and Mutations Associated with Amelanotic Melanoma

It has been observed that amelanotic melanoma occurs often in patients who are older than those with pigmented melanoma (PM) and is often linked to characteristics such as red hair, fair skin, an abundance of freckles, and sun-sensitive phenotypic indices [13,14]. The following traits indicate that the gene variants might also be linked to the development of AM. Moreover, contrary to previous assumptions, amelanotic melanoma cells retain the ability to produce melanin, as demonstrated by the expression of tyrosinase and MITF. This suggests a connection between AM and reduced activity of enzymes responsible for melanin production, such as tyrosinase (TYR) [15]. Research suggests that uncommon genetic mutations in pigmentation genes, such as TYR and oculocutaneous albinism II (OCA2), may play a role in susceptibility to amelanotic melanoma. It has been hypothesized that loss of function in somatic cells contributes to the loss of tumor pigmentation. Studies have reported a higher rate of somatic mutations in these genes in AM than in PM. The OCA2 variant associated with a higher prevalence of AM was p.V443I. The rare variants in TYR identified in the AM population were p.A23T, p.T373K, and p.P460L. The p.A23T variant showed the most considerable distinction between AM and PM frequencies [14]. In another study, the TYR variant associated with higher AM occurrence was R402Q [13]. Melanocortin 1 receptor (MC1R) serves as a genetic determinant of the aforementioned phenotype, particularly in relation to freckling and red hair [16]. The dominant allele of MC1R was more prevalent in patients with AM, as the MC1R R/R genotype occurred nearly three times more frequently in the AM population than in the PM group. The MC1R r/r genotype was also more common in the AM group. Furthermore, the methylthioadenosine phosphorylase (MTAP) protective allele, which is strongly associated with lighter skin, was found to be more prevalent in the AM group, in contrast to the Patatin-like phospholipase domain-containing protein 9 (PLA2G6) protective allele [13]. Another gene associated with AM is MITF, a critical component in the differentiation, survival, and progression of melanocytes, as well as in the pathogenesis of melanoma. In addition, MITF regulates melanin production and cell survival pathways and plays a dual role in promoting melanoma progression while also inhibiting invasion [17,18]. The presence of the MITF E318K mutation is linked to an enhanced likelihood of melanoma development and is accompanied by certain distinctive phenotypic features, including fair complexion and freckles. A genetic component associated with the MITF E318K allele and the occurrence of AM has been suggested [19]. Other genes that may potentially be linked to the occurrence of AM include tyrosine protein kinase (KIT), cyclin-dependent kinase inhibitor 2A (CDKN2A), and the v-raf murine sarcoma viral oncogene homolog B1 (BRAF) V600E mutation [20,21]. Table 1 summarizes genetic alterations and mutations associated with AM.

### 2.3. Differences in Gene Expression Profiles Compared to Pigmented Melanoma

In addition to the previously mentioned genes, further research is being conducted to explore additional differences in expression among these melanomas. The establishment of a comprehensive comparison of gene expression profiles between AM and PM would be beneficial for the diagnosis and development of personalized treatments. Researchers have utilized single-cell RNA sequencing (scRNA-seq) to identify distinct cell clusters exhibiting varying gene expression patterns related to melanin synthesis and cell cycle regulation following immunotherapy treatment. Cells with pigmentation demonstrated the presence of melanin granules expressing Melan-A, also known as the melanoma antigen recognized by T cells (MART-1), a protein derived from the MLANA gene, and Human Melanoma Black (HMB45), an antibody that specifically identifies the premelanosome protein (Pmel 17) antigen. In contrast, the amelanotic cells did not express HMB45. The expression of genes related to melanin synthesis, such as MITF, MLANA, PMEL, and TYR, was found to be decreased in the amelanotic clusters, while the levels of genes associated with lysosomes and endosomes, including lysosomal-associated membrane protein genes (LAMP1, LAMP2), the Ras-related protein gene (RAB4A), and the mannose-6-phosphate receptor gene (M6PR), were found to be increased in these clusters compared to the pigmented cluster. In contrast, cell cycle genes, including retinoblastoma protein gene (RB1), proliferating cell nuclear antigen gene (PCNA), and cyclin-dependent kinases genes (CDK2, CDK4, CDK6), were upregulated in PM compared to AM. In addition, immunohistochemical staining results for antigen Kiel-67 (Ki-67), a marker of cell proliferation, revealed that the proliferative capacity of pigmented melanoma cells surpassed that of amelanotic cells [22]. On the contrary, another study demonstrated a higher Ki67 positivity index in AM, as well as an increased expression of cyclooxygenase 2 (COX-2). The elevated expression of COX-2 has been associated with the modulation of angiogenesis, cellular migration, invasion, proliferation, and apoptotic resistance, which together contribute to tumor progression [23]. The lower expression of MITF and CDK2 in AM has also been demonstrated by researchers using mass-spectrometry-based proteomics. In addition, this study showed a higher expression of proteins involved in cell migration and invasion in AM, such as neuroblast differentiation-associated protein (AHNAK), plectin (PLEC), alpha-actinin-4 (ACTN4), and galectin-1 (LGALS1). AHNAK, in particular, is associated with poor prognosis and metastasis through the activation of the transforming growth factor beta (TGFβ) signaling pathway and subsequent epithelial–mesenchymal transition (EMT) [8].

## 3. Biochemical Pathways Involved in Melanin Production

### 3.1. Overview of the Melanogenesis Pathway

**Figure 1:**
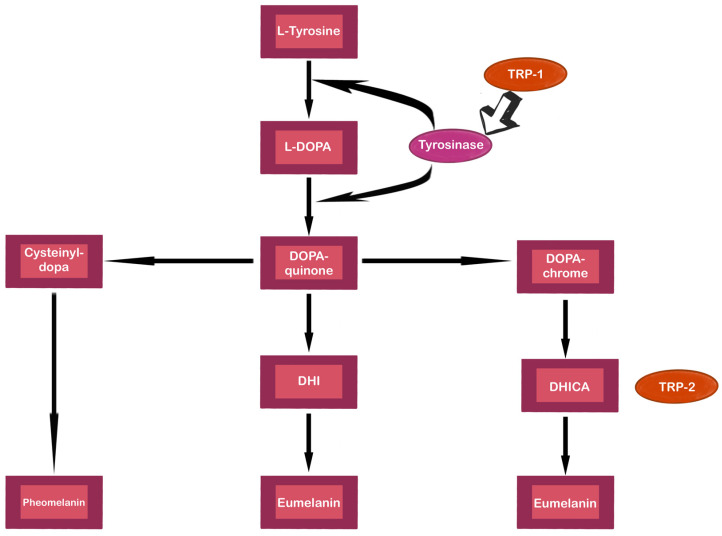
The schema of the melanogenesis pathway. Tyrosinase is an enzyme that catalyzes the conversion of L-tyrosine into L-DOPA. Tyrosinase-related protein 1 (TRP-1) and tyrosinase-related protein 2 (TRP-2) stabilize and increase the activity of TYR. Moreover, TRP-1 upturns the eumelanin vs. pheomelanin ratio. TRP-2 takes part in the tautomerization of DOPA chrome into DHI-2-carboxylic acid (DHICA). TRP-1 oxidases DHICA. The figure is based on the data of the studies [27,28,29].

Melanogenesis, the production of melanin, is a complex and intricately regulated process responsible for determining skin and hair color. Understanding the detailed mechanisms of melanogenesis, including the regulatory roles of various genes and epigenetic modifications, holds significant potential for the development of targeted therapies for pigmentation disorders [24,25,26]. An overview of melanogenesis is presented in Figure 1.

### 3.2. Key Enzymes and Cofactors Involved in Melanin Synthesis

Melanin production occurs within the epidermal melanin unit (EMU), where a single melanocyte connects with 10 to 36 keratinocytes via dendrites. During embryonic development, melanocytes originate from the neural crest cells in the dorsal neural tube and migrate along the dorsolateral pathway to the epidermis and hair follicles. Schwann cell precursors also contribute to the formation of melanocytes. Melanogenesis occurs within melanosomes, specialized intracellular organelles in melanocytes that mature through four stages from premelanosomes, resembling early/late endosome-like structures, to fully melanized organelles [30,31,32,33,34]. Important proteins involved in the formation of melanosomes include PMEL17, which aids in the polymerization of melanin and maintains the structure of melanosomes, and ocular albinism type 1 (OA1), a G-protein-coupled receptor that regulates the size and composition of melanosomes. The membrane-associated transporter protein (MATP), also known as solute carrier family 45 member 2 (SLC45A2), plays a crucial role in sorting tyrosinase and regulating melanosomal pH. Additionally, MART1 controls PMEL17 expression and stability. Two primary pathways are responsible for transporting proteins to melanosomes. One route is mediated by adaptor-related protein (AP) complexes, mainly AP-3 and AP-1, while the other is facilitated by the biogenesis of lysosomal organelle complexes 1 and 2 (BLOC-1 and BLOC-2). AP-3 transports tyrosinase, whereas AP-1 organizes tyrosinase and TYRP1 with a kinesin-like protein (KIF13A). BLOC-1 delivers ATPase Copper Transporting Alpha (ATP7A), which is crucial for tyrosinase activity [30,35,36]. Melanocytes transfer melanin-filled melanosomes to neighboring keratinocytes through mechanisms involving microtubule-associated motor proteins, such as kinesins and dyneins. Actin-based processes facilitated by RAB27A, melanophilin (MLPH), and myosin-Va (MYO5A) help dock melanosomes to the plasma membrane for transfer [37]. The critical transcription factor in melanogenesis is MITF, which acts as a transcriptional activator for several key melanogenesis-related genes, including TYR and tyrosinase-associated protein 2 (TYRP-2), also known as dopachrome tautomerase (DCT). In addition, MITF plays a crucial role in managing melanocyte growth and survival by influencing the levels of p16INK4A and p21CIP1, which are cyclin-dependent kinase inhibitors [35,38,39]. MITF also helps regulate melanosome distribution and their transfer to keratinocytes by controlling the expression of RAB27A [30,40].

### 3.3. Regulation of Melanin Production by Cellular Signaling Pathways

Tyrosinase, a membrane-bound glycoprotein, acts as the primary initiator of melanin synthesis by converting L-tyrosine into DOPA and then into dopaquinone. Dopaquinone is highly reactive and follows two reaction chains to produce a brown-black eumelanin or a yellow-red pheomelanin. Eumelanin production involves a series of intramolecular cyclization reactions that result in the formation of leukodopachrome (L-cycloDOPA) from dopaquinone. Subsequently, redox exchange occurs to produce dopachrome and DOPA. Dopachrome can either form 5,6-dihydroxyindole-2-carboxylic acid (DHICA) through TYRP2 and eumelanin via TYRP1 or convert it into 5,6-dihydroxyindole (DHI), followed by eumelanin, which involves TYR. For pheomelanin production, dopaquinone, in the presence of cysteine or glutathione, converts to 5-S-cysteinyldopa (5SCD) or glutathionyldopa, which then forms quinoline and polymerizes into pheomelanin [30,41]. The transcriptional upregulation of melanogenic genes is largely attributed to the activation of MITF through the cyclic adenosine monophosphate (cAMP) pathway. This pathway is initiated by mitogen-activated protein kinase (MAPK) extracellular signal-regulated kinase (ERK) signaling and ribosomal S6 kinases, which are downstream of KIT or MC1R. The process of MC1R activation results in an increment of cAMP levels through the activation of G-protein-coupled receptors (GPCRs). cAMP activates protein kinase A (PKA), which phosphorylates cAMP response element-binding protein (CREB), subsequently enhancing MITF expression. Moreover, MITF targets a specific sequence in the tyrosinase gene, leading to a modest upregulation of tyrosinase in response to alpha-melanocyte-stimulating hormone (α-MSH), a peptide derived from proopiomelanocortin (POMC). Other POMC-derived peptides, such as β-MSH and adrenocorticotropic hormone (ACTH), similarly promote melanogenesis by utilizing the same pathway. Additional G-protein-coupled receptor pathways include corticotropin-releasing factor (CRF), endothelin, wingless-related integration site (Wnt)/β-catenin, adrenergic, glutamatergic, and skin opsins signaling pathways. Additionally, receptor tyrosine kinase pathways, such as stem cell factor (SCF)/KIT, basic fibroblast growth factor (bFGF), hepatocyte growth factor (HGF), neuregulin 1 (NRG1), and bone morphogenetic protein (BMP) pathways, activate melanogenesis via MAPK pathway activation and MITF expression [35,42,43]. Forskolin, a compound that increases cAMP levels, increases MITF protein levels without affecting tyrosinase mRNA, suggesting post-transcriptional regulation. α-MSH also upregulates TYRP2, while agouti signaling protein (ASIP) decreases its expression [43]. Furthermore, antioxidant defense systems, such as the thioredoxin and glutathione systems, have been found to regulate melanogenesis. Reduced thioredoxin (Trx) has been shown to suppress melanin synthesis by reacting with the binuclear copper center of tyrosinase and inhibiting its activity, while thioredoxin reductase 1 (TrxR1) levels correlate with melanin production, suggesting their roles in skin pigmentation regulation. Glutathione (GSH) is involved in melanogenesis through two mechanisms: direct interaction with tyrosinase’s active site, activating the enzyme at low GSH concentrations but inhibiting it at higher concentrations, and the reaction of the GSH thiol group with dopaquinone, leading to pheomelanin formation. The inhibition of GSH synthesis promotes tyrosinase activity and eumelanin formation [44].

## 4. Dysregulation of Melanogenesis in Amelanotic Melanoma

DCT and TYR are melanogenic enzymes and as melanosomal proteins share structural features [45]. Amelanotic cells are characterized by very low TYR protein levels [46,47]. TYR is kept as a partially glycosylated precursor in the endoplasmic reticulum and then is prematurely deteriorated by proteasome machinery. However, DCT has been shown to be an entirely processed and stable protein. DCT and TYR have separate ER exit signals and, therefore, a distinct maturation and solidity along the amelanotic secretory pathway [48]. TYRP1 mutations have been detected in some patients with AM [14]. MITF, melanocortin 1 receptor (MC1R), and p14ARF mutations can be present [1,16]. MITF, commonly expressed in melanomas, plays a major part in transformation and progression, as it regulates the progression of TYR [49,50]. MC1R genotypes are associated with red hair color [10]. MYC proto-oncogene with 8q24 copy number gains may participate. A study showed a connection between high 8q24 copy number gains and increased cytoplasmic and membranous c-MYC and decreased MITF protein expression, which correlates with decreased TYR expression [1,50]. Cyclin-dependent kinase 2 (CDK2), a regulator of cell cycle progression, is downregulated [8]. The expression of CDK2 in melanocytes is regulated by MC1R [51]. Studies have shown different views on whether BRAF and KIT mutations are present in AM [1,16].

## 5. Molecular Induction Pathways in Amelanotic Melanoma

### 5.1. Role of Oncogenic Signaling Pathways (e.g., MAPK, PI3K/Akt) in Tumor Initiation and Progression

**Figure 2:**
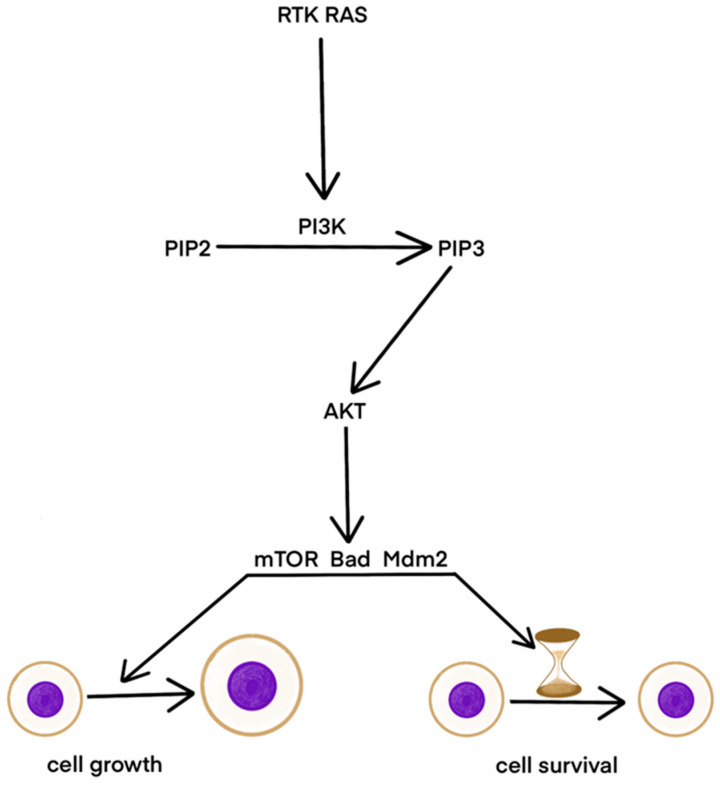
The role of the PI3K/AKT pathway in AM. RTK and RAS induce PI3K, which triggers PIP2 to PIP3 transition. PIP3 activates through AKT mTOR, Bad, and MdM2. These factors cause cell growth and survival.

The development of cutaneous melanoma is often the result of mutations in signaling pathways critical for cell survival, such as the MAPK and PI3K/AKT pathways. The MAPK pathway regulates cell growth, proliferation, differentiation, and apoptosis. Mutations in this pathway lead to excessive signal activation and uncontrolled cell growth. Activation occurs through the binding of a growth factor to receptor tyrosine kinase (RTK), triggering the RAF, MEK, and ERK cascade, which activates transcription factors. The PI3K/AKT pathway also influences cell growth and proliferation. PI3K, activated by RTK or RAS, phosphorylates PIP2 to PIP3, which activates AKT. AKT promotes cell growth and survival through mTOR, Bad, and Mdm2 (Figure 2) [52].

### 5.2. Dysregulation of Cell Cycle Control and Apoptosis Pathways

Amelanotic melanoma cells undergo a reduced ability to undergo spontaneous apoptosis while becoming melanotic. In the melanotic lineage, approximately 30% occur in the S+G2/M phases, of which approximately 33% undergo apoptosis. The slower growth of this melanoma line is due to its primary proliferative and apoptosis-related activity. Expansive growth of the melanotic lineage is based on reduced capacity for spontaneous apoptosis, especially in S+G2/M cells. A melanotic to amelanotic transition will occur with consequences in the regulation of cell termination and death. Understanding these programs is crucial to developing effective therapies that address the amelanotic features of the melanoma they contain [53].

### 5.3. Immune Evasion Mechanisms Employed by Melanoma Cells

**Figure 3:**
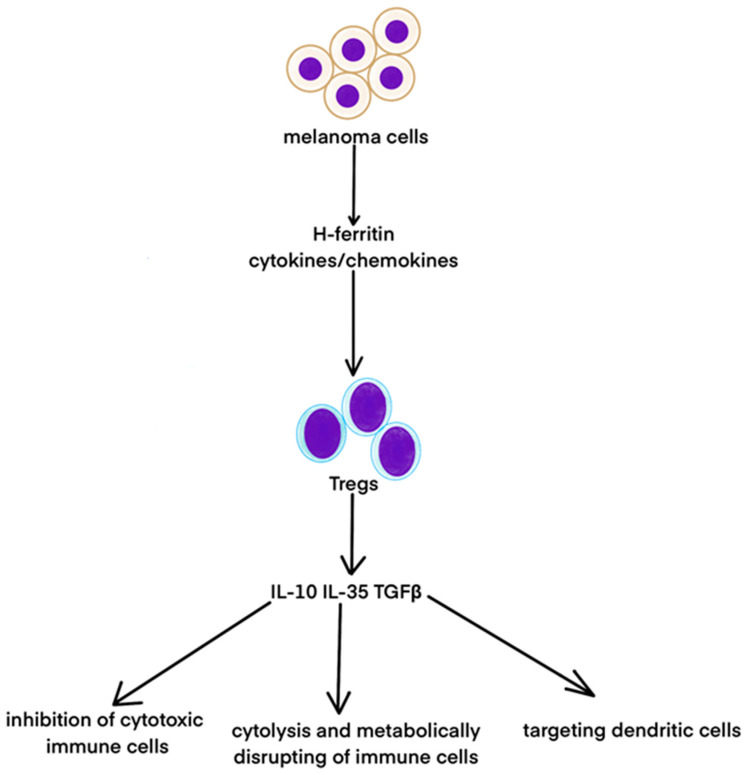
The role of Tregs in immune system suppression in AM.

Regulatory CD4+ T cells (Tregs) actively control excessive immune responses to host damage. Tumor cells exploit these protective functions to evade immunity. In both amelanotic and pigmented melanoma, Treg numbers increase in peripheral blood, lymph nodes, and the tumor microenvironment, reducing the cytolytic function of anti-tumor immune cells. Melanoma recruits/induces Tregs by secreting H-ferritin and cytokines/chemokines that modulate Treg function in the tumor microenvironment. Tregs suppress the immune system through four mechanisms: releasing immunosuppressive cytokines IL-10, IL-35, and TGF-β, which inhibit cytotoxic immune cell activity, inducing cytolysis of immune cells, targeting dendritic cells (antigen-presenting cells) [54], and metabolically disrupting immune cell function (Figure 3) [55].

Melanoma cells secrete H-ferritin, cytokines, or chemokines, which activate Tregs to secrete IL-10, IL-35, and TGFβ. These factors lead to the inhibition of cytotoxic immune cells, cytolysis, and the metabolic disruption of immune cells and targeting dendritic cells.

Dysregulated antigen processing inhibits CD8+ T cells from recognizing tumor cell antigens. Effective T cell cytotoxicity requires antigen presentation by mature dendritic cells (DCs). Other cells, such as myeloid-derived suppressor cells (MDSCs) and regulatory T cells (Tregs), accumulate in the melanoma microenvironment, disrupting the balance between immune suppression and stimulation. MDSCs release reactive oxygen species (ROS), like nitric oxide (NO), inhibiting NK cells and T cells. Tregs produce IL-10 and indoleamine 2,3-dioxygenase (IDO), further suppressing NK cells and CD4+ and CD8+ lymphocytes. Melanoma cells evade NK cell recognition by shedding MICA/B, reducing interaction with the NKG2D receptor. Additionally, decreased argininosuccinate synthetase levels in melanoma cells lower arginine production, impairing T cell survival and proliferation. These mechanisms collectively contribute to defective immune recognition and influence melanoma prognosis [56].

## 6. Biomarkers and Diagnostic Approaches

### 6.1. Identification of Biomarkers for the Early Detection of Amelanotic Melanoma

Populational studies have shown that amelanotic melanoma is more difficult to diagnose than the pigmented form of a tumor, which correlates with the poorer survival rate of patients diagnosed with AM [2]. That is why it is crucial to find specific biomarkers that would enable quicker diagnosis. First, miRNAs are small non-coding RNAs that regulate genic expression and are often deregulated in cancerous cells. It has been established that they may be found in bodily fluids, such as plasma, serum, and whole blood; thus, their altered levels may be measured to establish a diagnosis [57]. Studies concerning levels of miR-221 in melanoma patients revealed that the molecules may be a useful biomarker in the diagnosis and prognosis of the tumor, as high levels of miR-221 correlated with poorer survival [58,59]. Similarly, the expression of miR-424 is also increased in melanoma patients. Higher levels of the molecule in the serum indicate decreased survival and are associated with a higher chance of metastasis [60]. In short, circulating miRNAs are promising biomarkers not only in the diagnosis but also in the prognosis of melanoma. However, more data are needed to find specific miRNAs for the amelanotic type of melanoma [61]. Next, circulating tumor-derived exosomes can also be a source of tumor biomarkers. In the case of melanoma S100B and Melanoma Inhibitory Activity (MIA), antigens are present in exosomes and may be used for diagnosis and prognosis of the disease. Higher MIA levels correspond with shorter survival of melanoma patients; nevertheless, further studies are needed to evaluate the role of exosome antigens in differentiation with other illnesses [62]. The proteins mentioned in this paragraph may also be present in the blood of melanoma patients; however, exosomal antigens are more sensitive. Another possible marker for melanoma found in the serum is galectin-3, a lectin released to serum by cancer and inflammatory cells. Galectin-3 has been proven to play a role in cell differentiation, adhesion, and metastasis; thus, it may be used as a valuable biomarker. However, closer attention needs to be paid to it in the future to fully understand its role [63].

### 6.2. Imaging Techniques and Molecular Profiling for Diagnosis and Staging

The diagnosis of AM, like any malignant tumor, is based on histopathological examination. Dermoscopy is a non-invasive, easily accessible method to visualize skin lesions. Although amelanotic melanoma may bring some challenges in diagnostics, this method demonstrates sensitivity and specificity of 61% and 90%, respectively [6].

Dermoscopy makes it possible to differ between benign lesions (such as hemangioma) and suspicious lesions that require biopsy for full histopathological evaluation. Dermoscopy can also differentiate melanoma from other malignant skin tumors.

In the evaluation of a skin lesion without the presence of pigmentation, attention should be paid to the presence of ulceration. In the absence of ulcerations and white lines, dermoscopy assessment should be based on the appearance of the blood vessels. The type of vessels (dots, lumps, straight lines, loop shaped, curved, serpentine, helical, or glomerular) and their arrangement (random, clustered in groups, snake shaped, linear, or centered) are taken into consideration. If numerous vessels of one type are arranged in a single pattern, they are called monomorphic; if there are different arrangements in the lesion, then it is called a polymorphic vascular pattern [64].

In addition to dermoscopy, high-frequency ultrasound may be found useful to assess the depth of the lesion [65]. Another method used for the identification of AMs is reflectance confocal microscopy (RCM), which is a great supplement to dermoscopy when the diagnosis is not certain. RCM enables the visualization of atypical networks, regression structures, and atypical vascular patterns of AMs in detail [66]. However, it is not widely used in everyday practice. The histopathology report should include information on the depth of tumor infiltration (Breslow scale), the presence of ulceration, and the completeness of resection [67].

Moreover, there are also radiological imaging techniques, such as PET/CT and CT. Positron emission tomography/computed tomography (PET/CT) is an imaging method using 18F-fluorodeoxyglucose that provides various valuable information, including metabolism, hypoxia, and proliferation. What is more, it is useful in the primary staging of melanoma and in monitoring treatment response [68]. A case reported by Nielsen et al. shows the importance of PET/CT in AMs, as primarily a lymph node metastasis from MM was found and PET/CT found a primary tumor in AM, which was hard to identify with the naked eye [69]. Similarly, another case was reported about a woman who was misdiagnosed with a verruca plantaris; however, PET/CT showed higher glucose metabolism, and a biopsy revealed it was actually AM [70]. Meanwhile, Joshi et al. have reported a case of AM, which masqueraded as adenocarcinoma of the rectum, and the final diagnosis was successful due to PET/CT and immunohistochemistry [71]. Taking the abovementioned case reports into consideration, PET/CT is an imaging technique worth considering during primal diagnosis and in finding metastatic areas. Likewise, an effective way of staging AM is computed tomography (CT), which is also a great tool for follow-up imaging [72]. Karnwal et al. have reported a case of cervical lymphadenopathy. A CT scan showed a metastatic mass in the biopsy that was confirmed by the diagnosis of AM [73]. Currently, imaging studies are recommended in patients with locally advanced melanoma or when metastases are suspected. Additionally, molecular profiling plays a key role in the diagnosis and staging of melanoma, as AM cells often exhibit distinct genetic mutations; for instance, in the BRAF, NRAS, and c-KIT genes. BRAF mutations are responsible for the activation of the MAPK/ERK signaling pathway, which is a key factor in cell proliferation and survival of melanoma cells. BRAF mutations are now the target of anti-melanoma therapy. Similarly, NRAS mutations also activate the MAPK pathway; however, in a different mechanism. Unlike melanoma with a BRAF mutation, NRAS mutations are more resilient to treatment. Finally, c-KIT mutation tests are advised to be run, as targeted therapy is also needed. In order to check the aforementioned mutations, new-generation sequencing may be found useful [74]. Other factors analyzed are CDKN2A, TP53, Neurofibromin 1 (NF-1), splicing factor 3b, subunit 1(SF3B1), programmed death-ligand 1 (PD-L1) [67].

### 6.3. Challenges in Distinguishing Amelanotic Melanoma from Other Skin Lesions

AMs are often difficult to diagnose and may be overlooked by both patients and doctors, so it is crucial to keep in mind that a seemingly benign lesion may be, in fact, melanoma. Any delay in time due to a wrong diagnosis may cost a person their life. Table 2 shows common misdiagnoses of AM.

## 7. Therapeutic Strategies for Amelanotic Melanoma

### 7.1. Targeted Therapies Against Oncogenic Signaling Pathways

Approximately 30–66% of melanoma cases, including AMs, are carriers of the BRAF gene mutation, which is a target of anti-melanoma therapy. BRAF inhibitors (BRAFi) include vemurafenib, dabrafenib, and encorafenib. The role of BRAFi is the inhibition of the MAPK pathway and, as a result, the tumors shrunk; however, the outcomes were not satisfactory. The response to BRAFi was short, as the MAPK pathway was being reactivated. In order to defeat MAPK signaling reactivation, MEK inhibitors are used, as they also inhibit MAPK. MEK inhibitors include trametinib, cobimetinib, and binimetinib [81,82]. The phase 3 clinical study performed by Long et al. proved that dabrafenib therapy in combination with trametinib improved 3-year OS compared with dabrafenib monotherapy, as the 3-year OS was 44% and 32%, respectively [83]. Even though BRAFi and MEK inhibitors suppress the growth of the tumor, the outcomes are still unsatisfactory; thus, it is recommended to incorporate immune checkpoint inhibitors into the anti-melanoma therapy, as tumors become immune to the standard therapy [82].

### 7.2. Immunotherapy Approaches for Enhancing Anti-Tumor Immune Response

Immunotherapy has revolutionized the treatment of various tumors, including melanoma. In normal circumstances, the immune system identifies and destroys improper cells; however, tumors have the ability to avoid being detected by the immune system. Immunotherapy drugs prevent melanoma from avoiding the immune response and enable tumor cells to be destroyed. CTLA-4 (cytotoxic T-lymphocyte-associated antigen-4) and PD-1 (programmed death-1) are immune checkpoints that negatively regulate T cell activity; therefore, it is possible to enhance immune reaction by blocking these checkpoints. The CTLA-4 inhibitor is called ipilimumab, and PD-1 inhibitors include nivolumab and pembrolizumab, which are often used in melanoma therapies and can be used synergistically, as CTLA-4 mainly suppresses immune response in lymph nodes, whereas PD-1 does so in peripheral tissues [84]. Clinical trials have shown significant improvement in survival in melanoma patients after using nivolumab and ipilimumab therapy versus therapies consisting of only one drug. The overall survival at 5 years was 52% in the nivolumab-plus-ipilimumab group, while in the nivolumab group, it was 44%, and it was 26% in the ipilimumab group [85]. Other studies also show the beneficial synergetic action of joined immunotherapy, which is rather well tolerated by patients with few adverse reactions such as colitis, diarrhea, and fatigue [86,87,88].

### 7.3. Combination Therapies to Overcome Resistance and Improve Treatment Outcomes

Resistance to targeted therapy and immunotherapy creates many problems while fighting against melanoma, so researchers try to overcome them. One of the methods is to target different points and combine drugs using different mechanisms; the outcomes of which are mentioned above. The other methods use alternative methods, such as combining fecal microbiota transplantation (FMT) with PD-1 inhibitors. FMT with anti-PD-1 resulted in a response rate of 65% with a 20% complete response. The method is considered to be safe for the patient; however, future research is still needed to fully understand the mechanisms behind it [89]. Another way to overcome resistance to therapy may be using vidutolimod, a CpG-A Toll-like receptor 9 agonist combined with anti-PD-1. Vidutolimod induces T-lymphocytes to destroy tumor cells by triggering an IFN response. This combination of drugs was effective in 25% of patients who had durable responses [90]. Lastly, an immune-modulatory vaccine against indoleamine 2,3-dioxygenase (IDO) and PD ligand 1 (PD-L1) combined with nivolumab also shows promising results with an ORR of 80% [91].

Another combination therapy that proves to be more effective than monotherapy is the combination of CTLA-4 inhibitors and PD-1 inhibitors. The study performed by Larkin et al. showed that the 5-year OS in advanced melanoma patients was 52%, and the ORR was 58% [85], whereas another study showed that the 6.5-year median OS was 72.1 months with good drug tolerance [86]. The next beneficial combination of two immunotherapeutic drugs is the usage of LAG-3 inhibitors with PD-1 inhibitors. A single usage of relatilimab and nivolumab in patients with advanced melanoma showed a prolonged survival by 5.5 months compared to nivolumab monotherapy [92].

Another promising combined therapy uses cancer vaccines with immune checkpoint inhibitors. The UV1 vaccine is made of three synthetic peptides that may induce anti-tumor activity, especially when combined with immunotherapy, such as ipilimumab. Studies have shown that such a therapy shows no additional toxicity, while ORR levels were 33%, and the 5-year OS was 50% [93,94]. Similarly, the NEO-PV-01 vaccine combined with nivolumab induces T cells to kill melanoma cells. Ott et al. have shown that the treatment resulted in an ORR of 59% and a 1-year OS rate of 96%, with good tolerance to therapy [95]. A phase I trial has proved that combining TIL adoptive cell therapy with nivolumab may be feasible and well tolerable for patients with metastatic melanoma. The ORR was 36% and the OS was 23 months [96].

## 8. Experimental Models and Preclinical Studies

### 8.1. In Vitro Models for Studying Molecular Pathways in Amelanotic Melanoma

Researchers have leveraged in vitro models to elucidate the key biochemical and genetic pathways involved in the development and progression of amelanotic melanomas, aiming to better understand the molecular underpinnings of this challenging disease [9,10]. Previous studies have shown that there are a few in vitro models used to examine amelanotic melanoma pathways such as genetically modified cell lines, three-dimensional culture models, and patient-derived xenografts [97,98,99]. Amelanotic melanomas are particularly challenging due to their heightened genomic instability, adaptive mechanisms, and resistance to conventional therapies [99]. It has been found that the pleiotropic anti-cancer activity of minocycline and doxycycline works against amelanotic melanoma cells. Based on the overall findings, it could be inferred that doxycycline exhibited greater potency compared to minocycline. The combination treatment resulted in changes to the cell cycle profile and reduced intracellular levels of thiol antioxidants, as well as mitochondrial membrane potential in amelanotic melanoma cells. Exposure of A375 and C32 cells to minocycline and doxycycline resulted in cytochrome c release and the activation of initiator and effector caspases. The anti-melanoma effect of the studied drugs seemed to involve the upregulation of extracellular signal-related kinases 1 and 2 (ERK1/2) and melanocyte-inducing transcription factor (MITF) pathways. Furthermore, an increase was observed in the level LC3A/B—an autophagy marker—upon treatment with minocycline or doxycycline in A375 cells [100]. Promising evidence of the combined effect of carvedilol and sorafenib on melanoma cells has been indicated, indicating a significant reduction in cell growth compared to the control. However, except for the combination carvedilol (2.5; 5 lM) and sorafenib (1; 5 lM), which did not show this reduction in cell growth, the co-treatment of the two drugs at 5 μM concentration was more effective in inhibiting cell viability compared to the individual effects of either drug in the Cit 32 cell line cultures. Additionally, it was observed that carvedilol and sorafenib affected interleukin-8 (IL-8) expression at the protein level in interleukin-1β-stimulated (IL-1β-stimulated) A2058 and C32 cell lines [101]. A recent investigation suggested that combining the local application of ketoprofen with UVA irradiation may offer a supportive approach to treating melanoma, potentially reducing the risk of cancer recurrence and metastasis. The combined treatment of 1.0 mM ketoprofen and UVA irradiation disrupted the normal state of C32 melanoma cells, leading to a decline in their viability. In contrast to C32 cells, melanocytes exhibited low sensitivity to both ketoprofen and UVA radiation, indicating a selective mode of action targeting melanoma cells. The combined treatment led to cytotoxic, anti-proliferative, and pro-apoptotic effects on C32 cells, which stimulated DNA fragmentation and altered the cell cycle [102]. Furthermore, it has been suggested that induced melanization can elicit varying responses, potentially triggering apoptosis through cytotoxic mechanisms (Ab cells) or exhibiting negligible impacts on cellular biology (B16F10) [103]. The varying sensitivity of amelanotic Ab melanoma cells to three proteasome inhibitors with distinct chemical structures has been established. Among these, epoxomicin, a peptide epoxyketone natural product from the Actinomycetes strain Q996-17, proved particularly effective. The research findings indicate that amelanotic Ab melanoma cells primarily undergo apoptosis through the mitochondrial signaling cascade but also exhibit some caspase-independent apoptotic mechanisms. Apoptotic signaling is so robust that it induces cell death without preceding cell cycle arrest, despite the stabilization of the cell cycle regulator cyclin-dependent kinase inhibitor (p21Cip1/Waf1) and a multifunctional cyclin-dependent kinase inhibitor (p27Kip1). The study further revealed that the accumulation of heat shock proteins (HSPs) fails to provide protection for amelanotic Ab melanoma cells against the cytotoxic impact of epoxomicin. Consequently, the distinctive sensitivity of amelanotic melanoma to epoxomicin warrants further investigation [53]. Previous studies have investigated that A375 and SKMEL28 cell lines displayed faster migration when compared with pigmented cell lines, such as MNT1 and Me290. It has been established that A375 has the fastest migration capacity among melanotic and amelanotic cell lines [104]. The lack of pigmentation in the A375 cell line could indicate a disruption in the pigmentation process associated with melanoma progression. Regarding the SKMEL28 cell line, its migratory capacity exceeded that of the MNT1 line, and despite being MITF positive, the absence of pigmentation is attributed to the presence of immature stage I and II melanosomes [105]. SKMEL28 cells exhibited a substantial set of pigmentation-related proteins, yet the lack of pigmentation may be attributable to transformations during tumorigenesis. Regarding proteins that could be predictive of migratory capacity or related to the amelanotic state of A375 and SKMEL28, 42 proteins were significantly upregulated in both amelanotic cell lines compared to MNT1 cells, suggesting shared characteristics, as evident from the PCA plot. This indicates that the proteome profile of SKMEL28 falls between the profiles of MNT1 cells and A375. The investigation revealed that SKMEL28, A375, and SKMEL23 cell lines demonstrate elevated migratory capacity and share upregulated proteins, such as AHNAK (neuroblast differentiation-associated protein), MCAM (melanoma cell adhesion molecule), and HMGA2 (high-mobility group AT-hook 2), which contribute to their aggressive phenotype. Conversely, MNT1 and Me290 cells exhibited lower levels of these proteins. Additionally, key proteins, like ITGA6 (integrin alpha-6) and EPHA2 (ephrin type-A receptor 2), were identified as playing integral roles in focal adhesion and disease progression. The upregulation of these proteins in amelanotic melanoma cell lines suggests a distinct, cancer-specific profile associated with epithelial–mesenchymal transition and heightened metastatic potential, underscoring the heterogeneity of melanoma. Previous research connected with the influence of hypoxia on the melanoma cells’ proteome showed that A375 cells exhibited the greatest HIF1a levels in both hypoxic and normoxic conditions, likely due to constitutive expression. Furthermore, CTSD (Cathepsin D), associated with increased aggressiveness, was elevated in hypoxia, particularly in MNT1 and SKMEL28 cells. Additionally, in hypoxia, 42 proteins were significantly upregulated in amelanotic cells, including LGALS1 (galectin-1), PLEC (plectin), and AHNAK, suggesting their potential involvement in malignancy. Conversely, CDK2 (cyclin-dependent kinase 2) was downregulated in amelanotic cells but increased in slow-migrating pigmented cells. These findings underscore the proteomic distinctions between pigmented and amelanotic melanomas, offering potential biomarkers for predicting disease progression and metastatic risk [8]. Furthermore, the A375 cell line exhibited the most aggressive invasive behavior by distinctly penetrating into the dermal layer, while the SKMEL28 cell lines recapitulated the early stages of melanoma invasion to varying degrees. The study found that combining A375 and SKMEL28 melanoma cells led to increased production of immune-regulating and pro-angiogenic factors. Supernatants from these cell cultures promoted the growth of endothelial cells in vitro. Additionally, the A375 cells showed tissue contraction associated with higher TGF-β (transforming growth factor β) levels and α-SMA (α smooth muscle actin) expression, suggesting fibroblast differentiation into cancer-associated fibroblast-like cells and a transition from epithelial to mesenchymal characteristics, consistent with A375’s highly invasive nature [106]. Some authors have evaluated the impact of static magnetic fields. For instance, in vitro research examined the combined impact of flavones and static magnetic fields from permanent magnets on gene expression and antioxidant enzyme activity related to the body’s defense against antioxidants. The study found that baicalin and baicalein are likely to possess anti-cancer properties, as they interfere with redox balance in the C32 cell line while also enhancing the expression of genes associated with the antioxidant system [107]. Previous studies have shown that curcumin, a photosensitizing agent used in photodynamic therapy (PDT), may have utility in personalized treatment approaches. The most effective combination therapy involved simultaneous electroporation with curcumin, followed by PDT. This combined therapy exhibited varied effects on the metabolic activity of different cell lines, but the DNA fragmentation of normal cells was significantly lower. For amelanotic melanoma, the most effective therapeutic approach was a 3 h exposure to the natural compound curcumin, followed by the application of electrochemotherapy (ECT) and photodynamic therapy. The concurrent administration of electrochemotherapy and photodynamic therapy was determined to be a more potent treatment approach compared to the individual application of either modality. The high efficacy towards malignant cells demonstrates the potential of curcumin-aided PDT with ECT [108]. Prior studies have indicated that compound 15 (5,6-dihydroxyflavone) demonstrates potential anticancer properties. In vitro research has highlighted the flavonoid core with hydroxyl groups as a promising area for investigating drugs to address amelanotic melanoma [109]. It has been acknowledged that both laboratory and animal studies have shown that flavonoids may help to prevent cancer development and could impede the proliferation of cancer cells. It has been suggested that apigenin, luteolin, 5,6-dihydroxyflavone, and genistein have been shown to decrease the survival of amelanotic melanoma A375 and C32 cell lines through both intrinsic and extrinsic pathways of apoptosis. Furthermore, it is also suggested that the ribosomal s6 kinase (RSK) pathway plays a role in how melanoma cells react to cytotoxic levels of specific flavonoids, potentially laying the groundwork for investigating the simultaneous application of flavonoids and RSK kinase inhibitors [109]. Furthermore, it has been indicated that 28-O-propynoylbetulin (EB5) may have diverse effects on melanoma cells, but specifically in C32 cells, it could act as a potent inhibitor of cell growth. The results indicate that EB5 may have promising applications in diverse therapeutic strategies targeting melanoma [110]. Moreover, Jasione montana L. has demonstrated considerable cytotoxic and pro-apoptotic effects on the amelanotic melanoma C32 cell line, along with an influence on mitochondrial membrane potential and caspase activation. This study indicated that extracts of J. montana may have the potential to be formulated into novel topical treatments with anticancer properties [111].

### 8.2. In Vivo Models for Evaluating Therapeutic Efficacy and Toxicity

As far as we know, not much previous research has investigated in vivo models specifically connected with amelanotic melanoma. The field continues to rely on appropriate in vivo models, such as genetically engineered mouse models (GEMMs) [112], xenograft models [113], and patient-derived xenograft (PDX) models [114,115]. Recent studies have highlighted the potential of targeting anti-apoptotic proteins, such as induced myeloid leukemia cell differentiation protein (Mcl-1), as a promising approach for treating amelanotic melanomas harboring BRAF mutations [116]. Additionally, the discovery of genetic alterations in key signaling pathways, like the mitogen-activated protein kinase (MAPK) and phosphoinositide 3-kinase/protein kinase B (PI3K/Akt) cascades, has enabled the development of targeted molecular therapies [116]. Mcl-1 is overexpressed in human cancer, and MITF contributes to cell proliferation. Research investigated ciprofloxacin’s impact on microphthalmia-associated transcription factor/induced myeloid leukemia cell differentiationc (MITF/Mcl-1) proteins in C32 melanoma cells to elucidate the drug’s effects on viability, proliferation, and apoptosis. The study found that ciprofloxacin formed complexes with MITF and Mcl-1 proteins and reduced MITF expression but increased Mcl-1 levels. Additionally, it decreased cell viability, led to glutathione (GSH) depletion, induced apoptosis through intrinsic pathways causing DNA fragmentation, and caused arrest in the G2/M phase of the cell cycle [49]. A 2005 study explored the use of transgenic Tyr-RAS+ Ink4a/Arf−/− mice as a xenograft model for spontaneous uveal amelanotic melanoma. This study found that amelanotic uveal melanomas developing in Tyr-RAS+ Ink4a/Arf−/− mice displayed diverse histological features characteristic of uveal melanoma, including some resembling epithelial and mesenchymal neoplasms. Immunohistochemical analysis demonstrated the expression of Melan-A, S100 protein and neuron-specific enolase (NSE) in these tumors. Additionally, the tumors were observed to express mRNA transcripts encoding MITF protein, tyrosine-related proteins 1 and 2 (TYRP1, TYRP2), and the “silver” protein [113,114]. Researchers have reported the development of a genetically engineered mouse model (GEMM) of BRAFV600E-driven melanoma, whose tumors faithfully recapitulated the characteristics of human amelanotic/oligomelanotic malignant melanoma. This model faithfully reproduced key characteristics of the human disease, such as the absence or low levels of melanin pigmentation, accelerated tumor growth, and metastatic potential. The capacity to accurately model the biology of this uncommon subtype in a preclinical setting has been crucial in clarifying the pivotal role of mutant BRAF in the pathogenesis of melanoma [112].

### 8.3. Advancements in Personalized Medicine Approaches for Patient-Specific Treatment

Brachytherapy utilizing Pallladium-103 (103Pd) plaques for iris melanomas was found to result in decreased tumor vascularity, a darkened appearance of the tumor surface, and a reduction in tumor thickness. While ectropion uvea improved, corectopia occurred. Iris atrophy and cataracts were common complications but did not significantly impact vision. Neovascular glaucoma developed in one patient, but no other major radiation-related complications were observed. This treatment approach achieved complete tumor control over a mean 63-month period and preserved the pupil. Both clinical and ultrasonographic monitoring can be used to assess tumor regression following radiation therapy [117]. Other studies investigated the efficacy of brachytherapy as a standalone treatment or in combination with neoadjuvant photodynamic therapy for managing amelanotic choroidal melanoma. The findings indicated that administering photodynamic therapy as a neoadjuvant approach prior to brachytherapy led to a reduction in tumor thickness in 73.4% of cases. This, in turn, enabled a decrease in the toxic radiation effects on visual function, without compromising the overall disease control achieved through the treatment approach [118].

## 9. Clinical Management and Prognosis

### 9.1. Surgical and Non-Surgical Treatment Options for Amelanotic Melanoma

Surgical treatment involves a wide excision, with margins. The wideness of the excision is determined by the disease stage. Non-surgical treatment comprises targeted therapy, radiotherapy, checkpoint immunotherapy, or photodynamic therapy [119,120]. Moreover, there are non-surgical modalities such as cryotherapy, topical imiquimod, laser therapy, and curettage [121]. Photodynamic therapy (PDT) can be a significant treatment option for choroidal AM [120,122]. Initial tumor regression was observed in 88% of patients. However, 44% of these occur in recurrent disease [120]. Furthermore, AM is sensitive to hypericin PDT. Hypericin is an extract from *Hypercium perforatum* L., which acts as a photosensitizer [123,124]. PDT can be also applied before brachytherapy to reduce tumor thickness and minimize the treatment-related toxicity of brachytherapy. Nonetheless, individual PDT shows worse therapy outcomes than brachytherapy [125]. It is worth noting that nanoparticle drug delivery systems can improve the efficiency of PDT because they facilitate the accumulation of photosensitizers into tumor tissue [116]. Moreover, AM exhibited a heterogeneous response to PDT [118]. It is worth noting that AM can be also treated by radiotherapy (RTX). AM has greater efficiency of RTH than melanotic ones due to the lack of melanin, which can decrease the outcome of therapy. Therefore, patients with AM exhibit significantly longer survival time in comparison to melanotic ones [126]. Amelanotic tumors can be treated by small molecules, which mimic melanogenic substrates. The therapy is based on that these molecules can be transformed into small-molecule melanin metabolites that are toxic to tumor cells. For example, N-Acetyl-4SCAP (NAcCAP), which is a substrate of mushroom tyrosinase, found a use for melanoma cell cytotoxicity. Robins et al. synthesized NAcCAP analogs, which exhibit tyrosinase-independent cytotoxicity against an amelanotic SK-Mel-24 melanoma [127,128]. Nonetheless, NAcCAP administration may be difficult because a fairly large number of AMs grow in the lung [129]. DOPA is used in melanin synthesis; thus, boron-labeled DOPA was administered to tumor cells, where it was uptaken by tumor cells. Another interesting therapeutic purpose is a treatment based on the emission of α particles as a result of the adsorption of thermal neutrons by 0B1-para-boronophenylalanine hydrochloride (10B1-BPA). The traveling range of these particles is approximately equal to the diameter of a single cancer cell; thus, this therapeutic approach could destroy cancer cells, with minimal injury to adjacent tissue. In addition, affinity and binding are proportional to the rate of melanin synthesis. However, amelanotic melanoma exhibits a relatively lower accumulation of 10B1-BPA than high-melanin-producing melanoma, perhaps for the sake of the aforementioned reason [26].

### 9.2. Prognostic Factors Influencing Patient Outcomes

A plethora of factors contributes to worse AM patient outcomes, such as increased age, T4 tumor size, higher N-stage, metastasis, ulceration, mitotic rate, or Breslow thickness [1,130]. It is worth mentioning that no survival prevalence was perceived in chemotherapy, immunotherapy, or radiation therapy [130]. Another common factor that influences a patient’s outcome is vitiligo. It is associated with a better therapeutic response in patients. However, this effect was observed in patients with melanoma; thus, it is unclear if this appearance occurs in AM patients. Another problem is that it is difficult to note vitiligo in amelanotic melanoma due to a lack of melanin pigmentation. Moreover, some case studies have shown that vitiligo can occur in patients after nivolumab treatment [131]. The thickness of the tumor may have a significant influence on response to PDT. Turkoglu et al. have shown that amelanotic choroidal melanoma with a thickness of greater than 3.0 mm might have a lower response to PDT [122]. The diagnosis of amelanotic melanoma can be difficult because the lesion may have a patient’s skin color, and due to these issues, it can be more advanced at diagnosis [2,16,119,132]. Moreover, clinicians often underestimate lesions because they can be mistaken for benign melanocytic, non-melanocytic neoplasm, or dermatitis [8,12,133,134,135,136]. Thus, histological diagnosis is required for suspected melanoma lesions [137].

### 9.3. Emerging Strategies for the Surveillance and Management of Recurrent Diseases

AM demonstrates a higher index of recurrence than melanotic lesions and also lower overall 5-year survival after diagnosis [138,139]. The recurrence of atypical Spitz melanoma (AST), which is often amelanotic, can be managed by an excision. Another method is to re-excise a lesion with positive margins post-biopsy [140]. Mohs micrographic surgery (MMS) can be a successful treatment method for often amelanotic lentigo malignant melanoma (LMM), which improves the recurrence rate compared to other forms of therapy [121]. Anorectal melanoma is a rare melanoma, which can be amelanotic in 30–70% of histological cases. A local re-excision is a method applied in recurrent disease treatment. Moreover, in the literature on melanoma, incomplete resection may be the only risk factor for recurrent disease [141]. Moreover, postoperative adjuvant radiotherapy can be used to prevent the local recurrence of AM after surgery [142]. The recurrence of choroidal AM can be managed by follow up, and in case of recurrent disease PDT treatment or ruthenium, plaque brachytherapy may be involved [143].

## 10. Future Directions and Challenges

### 10.1. Unraveling the Complexity of Amelanotic Melanoma Heterogeneity

AM has been identified in all major histological subtypes [144]. AM can be assigned to several histological subtypes: superficial spreading, nodular, lentigo maligna, and acral lentiginous melanoma [2]. AM can be also divided into three groups: amelanotic, partially pigmented, and lightly colored melanoma. These groups were established on the basis of the presence and amount and melanin. Another division consists of the rate of tumor growth, superficial spreading, and nodular melanoma [12]. It is important to note that AM can be also presented as red skin lesions, which can be a diagnostic challenge because it can simulate pyogenic granuloma or hemangioma [9,21]. It is worth noting that nodular AM growth is faster in melanotic melanoma. AM can be also be differentiated based on vessel morphology, which is important because the correct diagnosis of AMs depends on vascular patterns [12,21]. Moreover, one of the major variants of melanoma lentigo malignant melanoma (LMM) can be almost or completely amelanotic, which is significant because it can result in misdiagnosis and the problem of margin definition [145]. The primary treatment method of LMM is surgical excision to achieve clear histological margins, which is difficult for the sake of the aforementioned purpose [121]. Furthermore, LMM is increasing faster than any other melanoma subtype; thus, these challenges may be significant in the future [146,147]. Furthermore, non-acral cutaneous melanoma (NACM) can be divided into several amelanotic subtypes: nodular and desmoplastic. Nodular melanoma can be presented as an amelanotic nodule thicker than other subtypes. An additional subtype is the desmoplastic subtype, which is marked by an amelanotic scar-like formation [148,149,150]. This lesion is histologically characterized by spindled melanocytes and abundant stroma [148]. Another important classification can be based on the presence of chromosomal aberrations. For example, there is a subgroup of melanomas with increased chromosomal copy number gains in 8q24 at MYC, which is characterized by amelanotic appearance [50].

### 10.2. Development of Targeted Therapies Based on Molecular Subtype Classification

AM is linked with the mutational activation of BRAF kinase and KIT, which can be used in targeted therapy for advanced-stage tumors [21]. Turner et al. have shown that all melanoma patients with BRAF fusions, especially AM (SEPT3-BRAF), respond to checkpoint inhibitor therapy [151]. Furthermore, the miRNA miR-204 was shown to inhibit AM cell motility by targeting AP1S, which gives therapeutic opportunities in BRAF-mutated melanoma [152]. Moreover, AM cell line A375 is sensitive to the BRAF inhibitor vemurafenib. Even if this cell line is resistant to vemurafenib, it can be treated by foretinib (MET inhibitor) and lapatinib (EGFR inhibitor) [153]. α-MSH, a ligand specific for MC1R, is overexpressed in both amelanotic and melanotic melanoma, which is significant because it might be used as a vehicle for targeted therapy and imaging [154,155,156]. Some studies have shown that the overexpressed of MC1R involves greater than 80% of melanotic and amelanotic human metastatic melanoma [155]. Desmoplatic melanoma (DM) is often amelanotic [157,158]. DM has exhibited frequent mutations, such as the deletion of mutations TSC1/TSC2 and the activation of NOTCH1 and KDR mutations. Therefore, these mutations present opportunities for targeted therapies [157]. Sidor-Kaczmarek et al. have shown that proteasome inhibition decreases the viability of Ab melanoma cells, which are amelanotic melanoma cells taken from the golden hamster Mesocricetus auratus Waterhouse breed in 1959. The most effective proteasome inhibitor was epoxomicin. Epoxomicin induces apoptosis in Ab melanoma cells on the mitochondrial pathway, partially by a caspase-independent way of apoptosis. However, it is not clear if this therapy method is efficient in the treatment of human amelanotic melanoma; thus, it requires more research [53].

### 10.3. Integration of Biomarkers and Imaging Techniques for Precision Medicine Approaches

Molecular biomarkers can help in AM staging [8]. Reflectance confocal microscopy (RCM) is an imaging method that has been successfully used to detect amelanotic melanoma with poor dermatoscopic features [159,160]. AM cells exhibit a higher expression of integrin alpha-6 (ITGA6) and, therefore, its higher expression can be associated with disease progression [8]. Another valuable method is immunohistochemical investigation, which is used to diagnosis melanomas, especially in amelanotic cases [161]. This method can be successfully used in the differentiation diagnosis of primary cervical malignant melanoma since it is able to classify AM. Moreover, this research staining combining S100 protein and HMB45 appears to offer the best result [162]. It is worth noting that targeting MC1-R byα-MSH can be used in radionuclide therapy and for diagnostic purposes [154]. The amelanotic or melanotic melanoma can be detected using imaging techniques, such as conjugating a near-infrared fluorescent dye. Nonetheless, amelanotic melanoma is often the most difficult to detect because it may exhibit lower MC1R expression. However, this issue may be overcome using a higher dosage of probe or instrumental optimization, or in some cases, more precision imaging methods, such as multispectral optoacoustic tomography [163].

## 11. Conclusions

### 11.1. Recap of the Key Findings Regarding Biochemical and Molecular Pathways in Amelanotic Melanoma

To better understand the molecular and genetic basis of AM, considering the phenotype of the affected patients may be of value. Patients with AM more often have certain features, such as fair complexion, freckles, and red hair [13,14]. Gene variants associated with some of these characteristics that are also more prevalent in AM include the dominant allele of MC1R [16], the MTAP protective allele [13], and the presence of the MITF E318K mutation [19]. AM exhibited elevated expression of proteins linked to cell migration and invasion, including AHNAK, PLEC, ACTN4, and LGALS1, with AHNAK notably associated with poor prognosis and metastasis [8].

### 11.2. Importance of Ongoing Research Efforts for Improving Diagnosis and Treatment

Contrary to previous assumptions, AM cells can produce melanin, as indicated by the expression of TYR and MITF, which suggests that reduced enzyme activity, particularly of TYR, may contribute to AM. Studies indicate that rare genetic mutations in pigmentation genes, such as TYR (p.A23T, p.T373K, p.P460L) and OCA2 (p.V443I), are more prevalent in AM than in PM [14,15]. Cancer vaccines combined with immunotherapy may induce anti-tumor activity, which may be used in therapy. The combinations involved a UV1 vaccine combined with ipilimumab or a NEO-PV-01 vaccine combined with nivolumab [93,94,95]. According to a phase I trial, TIL adoptive cell therapy combined with nivolumab might be used to treat metastatic melanoma patients [96].

### 11.3. Potential Impact on Patient Care and Outcomes in the Future

Single-cell RNA sequencing demonstrated a lack of HMB45 expression, a decrease in the expression of genes involved in melanin production (besides the aforementioned MITF and TYR, also MLANA and PMEL), and cell cycle genes (RB1, PCNA, cyclin-dependent kinases genes) but an upregulation in genes associated with lysosomes and endosomes present in AM [22]. The dysregulation of melanogenesis in AM is a more complex issue, as proteins interact with each other, regulating their expression [50,51]. As for molecular signaling pathways, in melanomas, the dysregulations of the MAPK and PI3K/AKT pathways are often present, both influencing cell growth and proliferation [52]. In the tumor environment, due to the secretion of H-ferritin, cytokines, and chemokines, regulatory CD4+ T cells accumulate. Tregs, along with MDSCs, release NO, IL-10, and IDO, further suppressing immune cells. Moreover, antigen presentation by the dendritic cells is impaired [55]. Due to the need for an effective and rapid diagnosis of AM, the role of biomarkers is becoming increasingly important. However, there is still a lack of biomarkers specific to AM. Generally, in both diagnosis and prognosis assessments for melanomas, miRNAs and exosomal antigens may be applied [59,60,62]. Dermoscopy, which facilitates AM detection based on vascular patterns [12], could be supplemented with reflectance confocal microscopy and PET/CT as a part of differential diagnosis in uncertain cases [66,70,71], such as those with high-frequency ultrasonography for assessing lesion depth [65]. Considering the tumor’s response to therapies, which varies based on its molecular environment, molecular profiling also plays a crucial role, and it is targeted mainly towards BRAF, NRAS, and c-KIT genes [74]. However, confusingly, studies have presented different views on the presence of BRAF and KIT mutations in AM [1]. Although BRAF inhibitors suppress tumor growth by inhibiting the MAPK pathway, their effectiveness is limited due to pathway reactivation. Thus, combining them with MAK inhibitors, which also work on MAPK, has shown enhanced outcomes; nevertheless, further improvements are needed [83]. Immune checkpoint inhibitors, i.e., drugs targeting CTLA-4 and PD-1, have shown significant improvements in survival rates, particularly when combined [85]. However, to overcome the resistance, other combinations that complement PD-1 with fecal microbiota transplantation [89], a CpG-A Toll-like receptor 9 agonist called vidutolimod [90] or immune-modulatory vaccines against IDO [91], are examined with potentially promising results. Apart from that clinical approach, research is carried out on many in vitro models exploring the impact of the experimental therapies on melanoma cells, mainly C32 cell lines. The disruption of the redox balance, the activation of caspases, or enhancing antioxidant mechanisms in C32 melanoma cells leads to decreased viability of the cells. In that context, anti-proliferative and pro-apoptotic effects were observed, among others, in studies with minocycline/doxycycline, UVA irradiation with ketoprofen, or flavonoids, both alone and combined with static magnetic fields [100,102,107,109]. Limited research has explored in vivo models specifically for amelanotic melanoma. According to two pieces on amelanotic melanoma of the eye, patients could preserve overall good visual function when treated with brachytherapy [117,118], which was possibly supplemented with neoadjuvant photodynamic therapy [118]. The overall 5-year survival rate following diagnosis is lower in AM compared to melanotic lesions [139]. The following factors are responsible for the worse outcomes of patients with AM: advancing age, larger T4 tumor size, higher N stage, the presence of metastasis, ulceration, elevated mitotic rate, or greater Breslow thickness [1,130]. The diagnosis is often delayed because of insufficient pigmentation [1] and resemblance to other dermatological conditions [9], including in more challenging cases, even red skin lesions [9]. Lentigo maligna melanoma (LMM), which can be almost or completely amelanotic and is increasing rapidly, consequently poses significant challenges in diagnosis and achieving clear surgical margins in excision [121,145]. However, due to the absence of melanin, radiotherapy could possibly be more effective in AM compared to melanotic melanoma [126]. Concomitant vitiligo has been suggested as another positive predictive factor [131].

## Figures and Tables

**Table 1:** Genetic alterations and mutations associated with AM.

Abbreviation of a Variant of a Gene	The Explanation of the Abbreviation
OCA2 p.V443I	oculocutaneous albinism II
TYR p.A23T	tyrosinase
TYR p.T373K	tyrosinase
TYR p.P460L	tyrosinase
TYR R402Q	tyrosinase
MC1R R/R	melanocortin 1 receptor
MC1R r/r	melanocortin 1 receptor
MTAP	methylthioadenosine phosphorylase
PLA2G6	patatin-like phospholipase domain-containing protein 9
MITF E318K	microphthalmia-associated transcription factor
KIT	tyrosine protein kinase
CDKN2A	cyclin-dependent kinase inhibitor 2A
BRAF V600E	v-raf murine sarcoma viral oncogene homolog B1

**Table 2:** Common misdiagnoses of AM.

dermal nevus	[75]
pyogenic granuloma	
eccrine poroma	
Kaposi’s sarcoma	
erythema nodosum	
non-healing ulcers	[76]
traumatic lesions	
benign odontogenic neoplasm	[77]
basal cell carcinoma	[78]
periorbital dermatitis	[79]
fungal infection	[80]
